# Short-Communication: A Comparison of the In Vitro Angiotensin-1-Converting Enzyme Inhibitory Capacity of Dairy and Plant Protein Supplements

**DOI:** 10.3390/nu9121352

**Published:** 2017-12-13

**Authors:** Carlotta Giromini, Ágnes A. Fekete, D. Ian Givens, Antonella Baldi, Julie A. Lovegrove

**Affiliations:** 1 Department of Health, Animal Science and Food Safety, University of Milan, Via Trentacoste, 2, 20134 Milan, Italy; carlotta.giromini@unimi.it (C.G.); antonella.baldi@unimi.it (A.B.); 2 Hugh Sinclair Unit of Human Nutrition Department of Food and Nutritional Sciences, University of Reading, Whiteknights, P.O. Box 226, Reading RG6 6AP, UK; a.afekete@gmail.com; 3 Institute for Food, Nutrition and Health, University of Reading, Reading RG6 6AP, UK; d.i.givens@reading.ac.uk; 4 Institute for Cardiovascular and Metabolic Research, University of Reading, Whiteknights, P.O. Box 226, Reading RG6 6AP, UK

**Keywords:** dairy protein, plant protein, in vitro gastro-intestinal digestion, ACE-1-inhibition

## Abstract

The consumption of supplements based on dairy or plant proteins may be associated with bioactive potential, including angiotensin-1-converting enzyme inhibitory (ACE-1i) activity, which is linked with blood pressure reduction in vivo. To gain insight into this proposed mechanism, the ACE-1i potential of protein-based supplements, including a selection of dairy (*n* = 10) and plant (*n* = 5) proteins were in vitro digested. The total digest was filtered and permeate and retentate were obtained. ACE-1i activity was measured as the ability of proteins (pre-digestion, ‘gastric’, permeate, and retentate) to decrease the hydrolysis of furanacroloyl-Phe-Glu-Glu (FAPGG) substrate for the ACE-1 enzyme. Permeate and retentate of dairy proteins exerted a significantly higher ACE-1i activity (mean of 10 proteins: 27.05 ± 0.2% and 20.7 ± 0.2%, respectively) compared with pre-digestion dairy proteins (16.7 ± 0.3%). Plant protein exhibited high ACE-1i in ‘gastric’ and retentate fractions (mean of five proteins: 54.9 ± 0.6% and 35.7 ± 0.6%, respectively). The comparison of the in vitro ACE-1i activity of dairy and plant proteins could provide valuable knowledge regarding their specific bioactivities, which could inform their use in the formulation of specific functional supplements that would require testing for blood pressure control in human randomly-controlled studies.

## 1. Introduction

Peptides derived from animal and plant proteins represent sources of potential health-promoting compounds and activities, such as angiotensin-I-converting enzyme inhibitory activity (ACE-1i) [1,2]. The mechanism of action of ACE-1 inhibition is not completely understood, but several studies have demonstrated that the inhibition of ACE can affect various regulatory systems of the body, such as the modulation of blood pressure (BP), the immune system, and central nervous system [3]. ACE-1 is a multifunctional enzyme that is localized in various tissues and associated with the renin-angiotensin system that controls BP. ACE-1 converts angiotensin I to the angiotensin II, which is a potent vasoconstrictor. Moreover, ACE-1 inactivates bradykinin, which has vasodilator activity [4]. ACE inhibitors exert anti-hypertensive effects through preventing bradykinin degradation and, thus, reducing angiotensin-II formation.

Protein-based supplements are frequently consumed for muscle gain in sports nutrition. Moreover, in spite of the lack of specific recommendation for sub-populations of older adults and/or hypertensive subjects, a growing body of evidence suggests that dietary proteins may allow blood pressure reducing activity and, therefore, should be tested in these target groups [5]. Among the ingredients used to prepare protein supplements, dairy and plant proteins are the most commercialized. Despite their nutritional role, dairy protein-based supplements contain ACE-1i peptides, which have been shown to reduce BP, particularly in pre-hypertensive subjects [6]. Although an emerging body of evidence supports the beneficial role of dairy protein consumption in lowering BP, the results of both human studies and in vitro studies investigating the potential anti-hypertensive effect of casein and whey-derived peptides are both limited and inconsistent. In particular, when the antihypertensive activity of peptides has been observed in vivo [6,7], the mechanism underlying this activity has been rarely determined. ACE-1i peptides were also identified in plant-based foods, including soybean, barley, wheat, and cherry [8,9]. To date, few in vivo or in vitro studies comparing the effect of dairy and plant proteins are available and the occurrence of ACE-1i compounds after gastro-intestinal digestion is not fully explored. Therefore, mechanistic studies are required to substantiate dairy and plant protein digestion patterns and their ACE-1i effects, to determine their true potential as ingredients or nutritional supplements for improving the functional value of dairy and plant-based foods. There is evidence that dairy and plant proteins contain encrypted bioactive peptides that can be released by digestion. Furthermore ACE-1i activity is related to native protein source and to the synergistic effect of the compounds released after their digestion, although to date a comparison of plant versus dairy ACE-1i activity after the different digestion phases has not been reported. Thus, the aim of the present study was to digest a selection of dairy and plant proteins in vitro to determine the ACE-1i activity at the different digestion phases.

## 2. Materials and Methods

### 2.1. Samples

The dairy protein-based supplements included were Volactiv Ultra Whey 80, ProCrisp, Denaturated Whey Protein Concentrate(WPC60), milk protein concentrate 85%, hydrolysed whey protein (low hydrolysis), hydrolysed whey protein (high hydrolysis), organic whey protein, Volactose whey permeate, whey protein isolate (WPI) (Volac International, Ltd., Orwell, Royston, Hertfordshire, UK) and calcium caseinate (Cambridge Commodities Ltd., Ely, Cambridgeshire, UK). The plant protein-based supplements included were wheat (ADM, Chicago, IL, USA), soy protein, soy protein isolate (My protein, Northwich, UK, https://www.myprotein.com), soy protein I, and pea protein (Pulsin, Gloucester, UK, https://www.pulsin.co.uk).

### 2.2. In Vitro Gastrointestinal Digestion (IVD)

The in vitro gastrointestinal digestion (IVD) of the dairy proteins (*n* = 10) and plant proteins (*n* = 5) was performed according to Mills et al. [10] with slight modifications [11,12]. Briefly, 20 g of each protein sample were mixed with 150 mL of distilled H_2_O in a plastic bag and maintained under agitation for 5 min. After 12 h of equilibration in the fridge, the samples were maintained at room temperature for 1 h The IVD procedure involved three phases. For the mouth phase 6.66 mg α-amylase in 2.1 mL of 1 mM CaCl_2_, pH 7 were added to the samples and an incubation of 30 min at 37 °C on a shaker was performed. The pH was then decreased to 2 with 6 M HCl. For the ‘gastric’ phase, 0.9 g of pepsin in 8.3 mL of 0.1 M HCl, pH 2. The samples were incubated for 120 min at 37 °C on a shaker. The pH was further increased to 7 with 6 M NaOH. For the small intestinal phase 0.2 mg pancreatin and 1.2 g bile in NaHCO_3_ 0.5 M were added to the samples before a final incubation for 180 min at 37 °C on a shaker. An aliquot of the pre-digestion and “gastric” fractions was sampled and snap frozen in liquid nitrogen before storing at −80 °C for further analysis.

### 2.3. Preparation of Peptide Extracts from Digested Samples

Subsequently, the digested samples were transferred to 3 kDa cut-off membrane (VIVASPIN 20 Sartorius, Göttingen, Germany). Each filter was previously activated with 0.1% Bovine Serum Albumin solution, followed by PBS solution. Samples were centrifuged for 16 min at 3500× *g*. The resulting permeate and retentate were aliquoted into Eppendorf tubes and snap frozen in liquid nitrogen before storing at −80 °C for further analysis. The ACE-1i effect—expressed as the percentage of ACE-1 inhibition—were measured in pre-digested, ‘gastric’, permeate, and retentate fractions.

### 2.4. ACE-1i Activity

The capacity of the protein fractions to inhibit ACE-1 activity was quantified using the ACE-i assay with furanacroloyl-Phe-Glu-Glu (FAPGG) as the synthetic substrate for the ACE-1 enzyme. The substrate solution (150 μL of 0.5 mM FAPGG substrate) and samples (10 μL of 0.2% w/w protein concentration) were incubated at 37 °C for 5 min in the microplate reader, according to XFluor4 software instructions (Tecan, Ltd, Männedorf, Switzerland). After the incubation, the ACE-1 enzyme (50 μL of 15 mU enzyme) was added to the sample/substrate mixture and the kinetic reaction started. The kinetic reaction was monitored for the following 30 min using the microplate reader at 340 nm with the use of captopril as a positive control [13]. Hydrolysis of FAPGG by ACE-1 enzyme resulted in a decrease in absorbance at 340 nm. A 100% ACE-1i activity would indicate complete inhibition of the enzyme. All experiments were performed in duplicate:

%ACE-1 inhibition = ((Absno sample − Abssample)/Absno sample) × 100.

Absno sample is the absorbance of the enzyme-substrate mixture in the absence of protein fraction, and Abssample is the absorbance of the enzyme-substrate mixture in the presence of protein fraction. ACE-1 enzyme, FAPGG and all other reagents were supplied from Sigma Aldrich (Dorset, UK).

### 2.5. Statistical Analysis

Each sample was analysed at least in duplicate and all experiments were replicated at least twice. The repeated-measure ANOVA test was performed using SPSS (SPSS software for Windows, release 11.0, SPSS Inc.) to determine the differences between the digestion phases in dairy and plant proteins (mean dairy and plant proteins). A *p* < 0.05 was considered as statistically significant. Relationships between ACE-1i in dairy and plant proteins were evaluated from the loading plots of principal component analysis (PCA), based on the correlation matrix consisting of four variables (pre-digestion, ‘gastric’, permeate, and retentate), using SPSS.

## 3. Results

Among the dairy proteins tested, the permeate of whey protein isolate (WPI) and hydrolysed whey protein (high hydrolysis) samples showed the highest ACE-1i capacity with values of 49.2 ± 0.64% and 48.3 ± 0.64%, respectively (Table 1). The permeate of denatured whey protein WPC60 exhibited the lowest ACE-1i capacity (3.8 ± 0.63%). WPC60, however, showed high percentage of ACE-1i in the retentate fraction. Furthermore, as for all the whey proteins tested, calcium caseinate contained higher ACE-1i compounds in the permeate fraction (23.9 ± 0.63%), compared with pre-digested calcium caseinate (18.1 ± 0.96%). All plant protein samples exhibited the highest ACE-1i in the ‘gastric’ fraction, ranging from 32.1 to 80.7%. The permeate fraction of plant proteins, however, showed the lowest percentage of ACE-1i. It was of note that the wheat protein sample showed an ACE-1i of 80.7 ± 1.4% for the ‘gastric’ fraction. This percentage decreased more than 10 times in the permeate fraction, after pancreatic digestion and filtration. Wheat protein, however, showed a higher percentage of ACE-1i in the retentate fraction, compared with the permeate.

**Figure 1:**
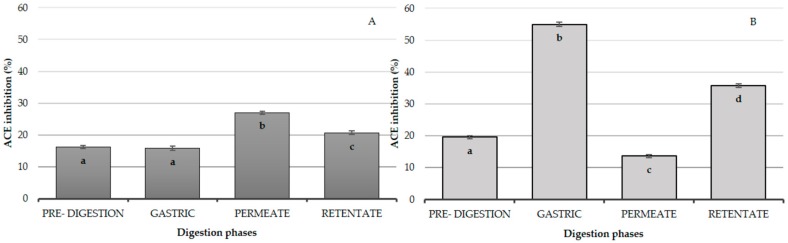
Effect of in vitro gastrointestinal digestion (IVD) on mean angiotensin-converting enzyme-1 inhibition (ACE-1i) capacity of the dairy proteins (*n* = 10, (**A**)) and plant proteins (*n* = 5, (**B**)). The bars represent the mean values at the different phases of the digestion, error bars represent SEM. Different superscript letters identify significant differences (*p* < 0.05) between bars.

Moreover, we found that mean values for permeate and retentate of dairy proteins (*n* = 10) exerted a significantly (*p* < 0.05) higher ACE-1i activity (27.05 ± 0.2% and 20.7 ± 0.2%) compared with that of pre-digestion dairy proteins (16.7 ± 0.3%) (Figure 1A). Conversely, we observed an opposite trend for plant proteins (*n* = 5) ACE-1i. As showed in Figure 1B, the mean plant protein permeates exhibited the lowest ACE-1i activity (13.6 ± 0.5%), compared with pre-digestion, ‘gastric’, and retentate fractions. The ‘gastric’ fraction of plant proteins exhibited a significantly higher ACE-1i activity of 54.9 ± 0.63%, compared with pre-digestion and the permeate plant protein fractions. Furthermore, the retentate of plant proteins showed a significant (*p* < 0.05) ACE-1i activity (35.7 ± 0.6%).

**Figure 2:**
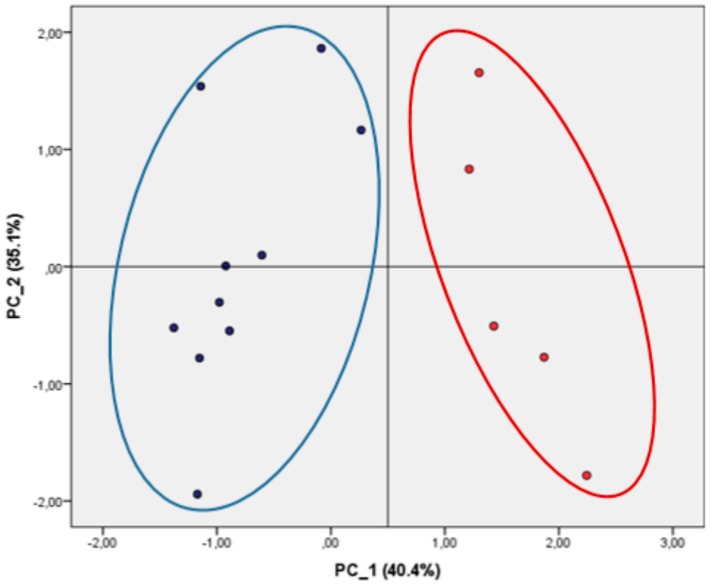
Principal component analysis score plot and clusters based on ACE-1i activity. Relationships between ACE-1i in dairy and plant proteins were evaluated from the loading plots of principal component analysis, based on the correlation matrix consisting of four variables (pre-digestion, ‘gastric’, permeate, and retentate). Dairy (blue points) and plant (red points) protein samples separate into two distinct groups. The score plots for the first two principal components PC_1 (40.4%) and PC_2 (35.1%) are indicated.

The different trend in ACE-1i activity observed in dairy and plant proteins was also confirmed by multivariate data analysis. As illustrated in Figure 2, Principal component analysis performed has shown a clear separation of dairy and plant proteins, according to their ACE-1i activity in the different digestion phases. The PCA was performed on all proteins samples (*n* = 15; 10 dairy and five plant based samples), which are described by four variables (pre-digestion, ‘gastric’, permeate, and retentate ACE-1i). In particular the relationships between ACE-1i in dairy and plant proteins were evaluated from the loading plots of PCA, based on the correlation matrix consisting of four variables (pre-digestion, ‘gastric’, permeate, and retentate). Dairy (blue points) and plant (red points) protein samples separate into two distinct groups.

## 4. Discussion

In this study, we measured ACE-1i activity of dairy and plant proteins in pre-digested protein fractions and after pepsin (‘gastric’), pancreatic digestion, and filtration (permeate and retentate fractions) with a 3 kDa membrane [14] to simulate the intestinal absorption. We used pepsin and pancreatin-bile to hydrolyse the proteins, representing the main proteolytic enzymes present in the stomach and small bowel during food digestion. Thus, the final hydrolysate obtained represented a pool of peptides resembling those generated during the physiological digestion of proteins in humans. These peptides could be absorbed by epithelial cells along the small intestine and enter the blood circulation or stay in the colonic environment. In the present study we reported that permeate and, to a lesser extent, retentate of whey-based proteins showed a significant ACE-1i activity compared with the pre-digestion whey-based proteins, showing that digestion influences the liberation of ACE-1i compounds. Furthermore, the permeate fraction of caseinate contained higher ACE-1i compounds, compared with the pre-digested protein. Caseinate, however, was the sole casein protein-based supplement sample included in the present study. Therefore, these data need to be confirmed by testing additional casein-based supplements. These data differ from those reported by Petrat-Melin et al. [15] who demonstrated a marked increase in ACE-1i of dairy proteins upon initial digestion (pepsin incubation), whereas at the end of pancreatic enzyme digestion they observed a smaller increase in inhibition. This difference may be the result of using varying digestion procedures and of the different food matrix tested. Additionally, Petrat-Melin et al. [15] used purified casein variants, whereas in the present study the digestion was performed on protein supplements.

IVD also liberated ACE-1i compounds from plant protein samples. Despite the high content of ACE-1i peptides in plant proteins, they remained predominantly in the retentate fraction after gastro-intestinal digestion. ACE-1i activity in the permeate fraction of plant proteins was more limited than the retentate fraction. However, an increase in ACE-1 inhibitory activity was observed after pepsin treatment, which suggests generation of significant numbers of bioactive ACE-1i peptides. These data are in agreement with those reported by Jimsheena et al. [16]. These authors investigated the ACE-inhibitory role of plant peptides derived from arachin (a peanut globulin) and observed that the degree of proteolysis and in vitro ACE inhibition of these peptides was highest after pepsin digestion; in contrast, the pancreatin digestion was not as effective [16]. Furthermore, Megìas et al. observed that the highest level of ACE-1 inhibition of sunflower protein was obtained after pepsin treatment [17].

The high bioavailability and digestibility [18] indicates that the dairy and plant proteins can be suitable for use as functional food-based supplements for reducing or preventing hypertension. However, their efficacy as anti-hypertensive supplements would need to be confirmed in human randomly-controlled dietary intervention studies. The results obtained have a number of implications. Initially, the potential beneficial effects of inclusion of these supplements in the diet of humans for anti-hypertensive effects. Furthermore, it provides evidence for the specific type of hydrolysis that is required for specific protein sources in order to obtain ACE-1i peptides. The later aspect can have important industrial application. In our experimental condition, we have shown that to obtain high concentrations of ACE-1i peptides the optimal hydrolysis treatment depends on the food sources, for dairy proteins pepsin-pancreatin treatment was optimal, whereas for plant proteins only pepsin treatment was required. Several authors reported ACE-1i activity in peptides produced from the in vitro digestion of protein, using a combination of physiological enzymes [15,19,20]. For peptides to be effective ACE-1 inhibitors, they must resist digestion and enter the circulation before reaching the target organ. Therefore, the ACE-1i activity observed in vitro does not directly translate into significant effects in vivo, due to differential bioavailability of peptides. The heterogeneity in individuals’ responsiveness to dairy and plant proteins can prevent identification of associations between dietary intakes and health, hinder the identification of health benefits for specific population groups and limit our understanding of the exact role of the different bioactives as ACE-1i. However, our recent study [6] has demonstrated a relationship between BP reduction in humans consuming supplements based on dairy proteins and the in vitro ACE-1i effect of the same protein samples tested in the human intervention study. Fekete et al. [6] investigated the effects of WPI and casein protein isolate supplements (45 g/day) for eight weeks on cardiovascular health, demonstrating a significant reduction in BP in both whey and casein protein-consumers, compared with the control group (no protein) in a double-blind cross-over RCT. The results reported in the present study support these data. In summary, we can speculate that bioactive peptides within dairy and plant proteins are in an inactive form within parent protein sequences with the requirement of hydrolysis for full ACE-1i activity to be realized. When absorbed, these hydrolysed peptides could reach their target tissues where they would contribute to ACE-1i effects. Our investigation has a limitation: the characterization of bioactive compounds in the different fractions (pre-digested, ‘gastric’, permeate, and retentate) was not performed, although a comprehensive number of highly-purified dairy (*n* = 10) and plant (*n* = 5) proteins were assessed after digestion, which closely mimicked the physiological in vivo digestion.

## 5. Conclusions

We demonstrated that dairy and plant based protein supplements were rich sources of bioactive peptides that had the ability to decrease ACE-1 activity, a key enzyme controlling BP in vivo. These data support the emerging body of evidence, which indicates the beneficial role of protein supplements in blood pressure modulation and indicates that a possible mechanism of action could be ACE-1i activity. Further research is needed to determine the overall bioavailability of both dairy and plant proteins after exposure to conditions that mimic the human gut. A characterization of the composition of the protein hydrolysate at all digestion phases, the elucidation of the relationship between the peptide structure and activity for their application as functionalized supplements await future studies.

## Figures and Tables

**Table 1:** Percentage of angiotensin converting enzyme inhibitory-1 activity after the in vitro digestion (IVD) phases. Results are presented as mean (standard error mean, SEM). ACE-1i activity was corrected for nitrogen content in all fractions tested.

Sample	ACE-1i (%)			
	Pre-Digestion	‘Gastric’	Permeate	Retentate
Dairy Proteins				
Volactiv Ultra Whey 80	15.08 (0.96)	16.58 (0.79)	18.40 (0.64)	14.50 (0.6)
ProCrisp	30.06 (0.96)	16.64 (0.79)	25.36 (0.64)	33.59 (0.6)
Denatured Whey Protein Concentrated (WPC60)	7.80 (0.96)	7.45 (0.79)	3.81 (0.64)	13.24 (0.6)
Milk Protein Concentrate 85	10.79 (0.96)	12.42 (0.79)	25.20 (0.64)	12.80 (0.6)
Hydrolysed whey protein (low hydrolysis)	11.46 (0.96)	15.76 (0.79)	27.76 (0.64)	21.08 (0.6)
Hydrolysed whey protein (high hydrolysis)	23.39 (0.96)	25.10 (0.79)	48.30 (0.64)	36.52 (0.6)
Organic whey protein	16.01 (0.96)	17.95 (0.79)	28.00 (0.64)	24.83 (0.6)
Volactose whey permeate	10.43 (0.96)	10.45 (0.79)	20.62 (0.64)	17.88 (0.6)
Whey protein isolate WPI	19.50 (0.96)	19.05 (0.79)	49.20 (0.64)	18.45 (0.6)
Caseinate	18.07 (0.96)	16.51 (0.79)	23.90 (0.64)	13.90 (0.6)
Plant Proteins				
Soy protein	16.28 (0.98)	37.06 (1.4)	10.40 (1.07)	63.31 (1.32)
Pea protein	36.28 (0.98)	58.56 (1.4)	28.03 (1.07)	23.85 (1.32)
Wheat protein	8.25 (0.98)	80.66 (1.4)	8.30 (1.07)	46.04 (1.32)
Soy protein I	22.11 (0.98)	67.08 (1.4)	10.95 (1.07)	24.57 (1.32)
Soy protein isolate	14.78 (0.98)	32.11 (1.4)	10.46 (1.07)	21.00 (1.32)
